# Nitrous Oxide Induced Posterior Cord Myelopathy: Beware of the Methyl Folate Trap

**DOI:** 10.7759/cureus.9319

**Published:** 2020-07-21

**Authors:** Dario A Marotta, Hassan Kesserwani

**Affiliations:** 1 Department of Research, Alabama College of Osteopathic Medicine, Dothan, USA; 2 Department of Neurology, Division of Neuropsychology, University of Alabama, Birmingham, USA; 3 Neurology, Flowers Medical Group, Dothan, USA

**Keywords:** nitrous oxide, myelopathy, methyl folate trap, neurotoxicity

## Abstract

Posterior cord myelopathy and subacute combined degeneration of the spinal cord are well-known complications of nitrous oxide abuse. Supplementation with vitamin B12 is an effective treatment strategy to correct low serum B12 levels or normal serum levels of dysfunctional vitamin B12 associated with this pathology. In this case, we report a patient with a one-year history of heavy nitrous oxide consumption; anywhere from eight to 30 canisters of 8 grams of nitrous oxide daily. The patient’s symptoms worsened after the institution of megadoses of vitamin B12, despite normal serum folate levels. This can be explained by the phenomenon of the methyl folate trap and warrants prompt supplementation with synthetic folate. We discuss the biochemistry of nitrous oxide toxicity and the underlying mechanisms contributing to the methyl folate trap.

## Introduction

Subacute combined degeneration of the spinal cord is a well-known complication of chronic nitrous oxide intoxication. It manifests as numbness of the upper and lower extremities, the Lhermitte phenomenon, with or without spasticity of the arms or legs, a stiff gait, or even signs and symptoms of a polyneuropathy. Behavioral and psychiatric manifestations are also variably described [1]. The radiological findings, including the inverted “V” sign of a posterior cord myelopathy, are well-known [2]. The biochemical lesions are well-described, including low or dysfunctional but normal serum B12 levels, as manifested by elevated homocysteine and methionine levels. Mutations of the methylenetetrahydrofolate reductase (MTHFR) enzyme leading to elevated homocysteine levels are also well-known and may predispose to nitrous oxide toxicity [3,4]. A much less known phenomenon is that of the methyl folate trap [5]. This phenomenon can occur in patients with subacute combined degeneration of the spinal cord when vitamin B12 supplementation is administered in the absence of folate administration, despite purportedly normal folate levels [6]. We describe exactly such a case, but instead one induced by nitrous oxide toxicity. We also aim to outline, in great detail, the biochemical lesions of nitrous oxide toxicity and the pathways underlying the methyl folate trap.

## Case presentation

A 41-year-old man was referred to the neurology clinic for a nerve conduction study for progressive bilateral upper and lower extremity paresthesias of one-month duration. The patient denied prior illicit drug use, but inexplicably reported he decided to start inhaling nitrous oxide. This developed into a daily habit. He bought whipped cream chargers in canisters from the internet and consumed anywhere from eight to 30 canisters per day. Each canister contains 8 grams of nitrous oxide. He emptied the canister into a balloon and inhaled the contents for one to two minutes, thereby sustaining a short-lived high for another one to two minutes. This continued for a duration of one year.

On one occasion, he underwent a dental procedure and noted that inhalational anesthesia with nitrous oxide was ineffective. He had developed a tolerance to nitrous oxide. Shortly thereafter, he woke up with a “tingly numb” sensation of his left arm and leg. Two weeks later, the “prickly tingling” sensation spread to his right arm and leg. His neck felt stiff. He developed a numb tongue and a sore feeling of his teeth. He developed numbness of his anterior abdomen and noted a sensation of butterflies in his stomach; almost described as hunger pangs. He felt he was in a fog, almost cloudy. He noted increasing forgetfulness. He was irritable and his temperament became volatile. He developed anxiety and depression. The patient noted fleeting waxing and waning of his vision; lasting seconds and without trigger. After one month of increasing symptoms, he quit inhaling nitrous oxide. He was referred to our clinic for a nerve conduction study due to his numbness. Upon triaging, we decided to evaluate him more thoroughly.

The patient’s past medical history was significant for hypertension, heartburn, and allergic rhinitis. He was a non-smoker. His medications included clonidine, atenolol, and montelukast. He was 5 feet and 5 inches tall, weighed 214 pounds, and had a body mass index of 35.6. His blood pressure was 163/106, pulse was 76, and temperature was 97.5 degrees Fahrenheit. His mood was irritable, but he was cognizant of the gravity of his situation. At times, he resisted questioning. His delayed recall was three out of three at two minutes. Digit span was seven out of seven. Verbal fluency for animals was at least 15 within one minute. Visuomotor skills as assessed by pantomime were preserved. A mini-mental status score was 30 out of 30.

His balance was preserved, with stable tandem walking. Romberg's sign was absent. Cranial nerve evaluation was normal throughout. Specifically, no ophthalmoplegia or facial weakness was noted. Visual acuity was 20/20 bilaterally. No discoloration was detected on Ishihara plates. His pupils reacted to light and accommodated briskly. A Marcus-Gunn pupil was absent. Funduscopic examination revealed sharp optic discs with preserved venous pulsations. Facial sensory was preserved to touch and sharp stimulus in all three divisions bilaterally. Taste was intact to salt and sugar. His speech was fluent with normal cadence and prosody. Motor examination revealed no weakness and all muscle groups graded 5/5 on the Medical Research Council Scale for Muscle Strength. No postural, intention or kinetic tremor was noted in the upper extremities. Lower extremity heel to shin was accurate bilaterally. Sensory examination to touch, pinprick, and joint position sense was preserved in the fingers and toes. No sensory level to pinprick or touch was noted anteriorly or posteriorly in the trunk. Vibration sense was mildly reduced in the toes to a tuning fork vibrating at 128 Hz. Reflexes including biceps, triceps, brachioradialis, knee and ankle jerks were surprisingly lively and symmetric.

Relevant serology was obtained. A summary of pertinent laboratory parameters is described in Table 1. Despite a normal serum vitamin B12 level, methylmalonic acid and homocysteine levels were elevated approximately five and three times above the upper normal limit, respectively. This implies dysfunctional vitamin B12 biochemistry; specifically, inhibition of the enzymes methionine synthetase (MS) and methylmalonyl coenzyme A mutase, respectively. Rapid plasma reagin (RPR), HIV serology, and neuromyelitis optica (NMO) IgG antibodies were all negative.

**Table 1:** Summary of patient serology The patient’s serology was significant for elevated levels of homocysteine (42.6 mcg/L) and methylmalonic acid (1926 nmol/L). H = Patient value above the upper limit of normal

Analyte	Patient value	Reference values
Hemoglobin	15.1	13.5-17.5 gM/dL
Hematocrit	42.7	38-50%
Vitamin B12	538	193-986 pg/mL
Mean corpuscular volume (MCV)	91.4	81.2-95.1 pg/mL
Folic acid	11.9	3.1-17.5 mcg/mL
Thyroid Stimulating Hormone (TSH)	1.08	0.358-3.740 mclU/mL
Homocysteine	42.6 H	0-14.5 mcg/L
Methylmalonic acid	1926 H	0-378 nmol/L
Copper	86	72-166 mcg/dL
Zinc	64	56-134 mcg/dL

**Figure 1:**
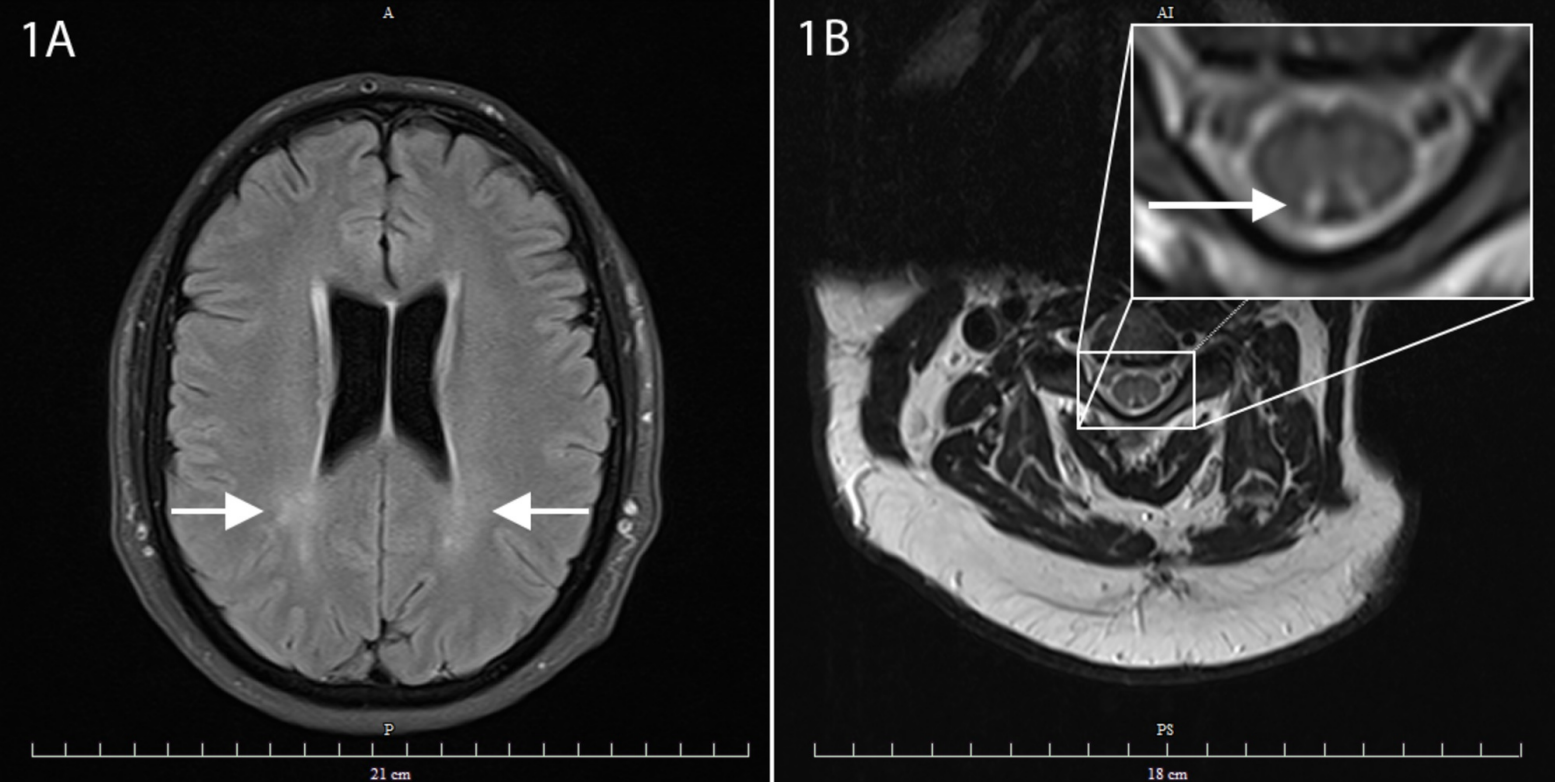
MRI of the neuraxis revealing signs of demyelination A: Axial MRI of the brain showing mild bilateral peri-atrial hazy demyelination without contrast enhancement (white arrows). B: Axial MRI of the cervical spine showing the classic inverted “V” sign suggestive of posterior cord demyelination (white arrow within inset). MRI = magnetic resonance imaging

MRI of the brain showed mild bilateral peri-atrial hazy demyelination without contrast enhancement (Figure 1A). MRI of the cervical spine revealed the classic inverted “V” sign suggestive of posterior cord demyelination (Figure 1B). These lesions did not enhance with contrast and were best visualized with the T2 weighted axial images at the levels of C3, C4, and C5. MRI of the thoracic spine was unremarkable. Due to the patient’s initial transient visual fluctuations, a visual evoked potential (VEP) was obtained and this revealed normal P100 latencies bilaterally, excluding any anterior visual conduction deficits. Due to the tongue numbness and teeth pain, a blink evoked potential was performed, and bilateral ipsilateral P1, ipsilateral P2, and contralateral P2 latencies were obtained. These latencies were within normal limits, excluding a fascicular trigeminal or central lesion of the principal sensory nucleus of the trigeminal nerve.

The patient was started on 5 mg megadoses of sublingual vitamin B12 daily. Four days later he started experiencing the Lhermitte phenomenon, which he described as a powerful tingling vibration that travelled down the arms, trunk, legs, and continued all the way down to the feet, elicited by neck flexion. The phenomenon was also elicited by elevating his arms and was prominent in the anterior abdomen. He further described it as an electrifying sensation. We immediately instituted large doses of folic acid supplementation at a dose of 4 mg daily and his diffuse numbness, including Lhermitte phenomenon, stopped worsening after five days, and may have improved slightly, but persisted. The patient continues on 5 mg mega doses of sublingual vitamin B12 and 4 mg folic acid daily. Noteworthy, his mood and mental fogginess had improved. His irritability dissipated and his mental clarity normalized after two weeks of high dose supplementation with both vitamin B12 and folic acid. We will continue to monitor him very closely, both clinically and with serum homocysteine and methylmalonic acid levels.

## Discussion

The complications of nitrous oxide neurotoxicity are well described in the literature. The most common complications include a myeloneuropathy, subacute combined degeneration of the spinal cord, and posterior cord myelopathy [1]. In order to understand nitrous oxide toxicity, one needs to understand the biochemistry of vitamin B12 (cobalamin). Cobalamin contains a cobalt ion with one valence electron which acts as a coenzyme for MS activity. MS converts homocysteine to methionine. Methionine is necessary for the formation of S-adenosyl methionine (SAM) which is necessary for the methylation of myelin sheath phospholipids. MS also demethylates methyltetrahydrofolate (MTHF) which provides a methyl group for the conversion of deoxyuridine to deoxythymidine. This reaction is essential for DNA replication and tissue proliferation. Nitrous oxide toxicity impairs cobalamin function by oxidizing the cobalt ion from the +1 to the +3 valence state and by inhibiting MS with the cascading effects listed above. Furthermore, nitrous oxide inhibits methylmalonyl coenzyme A conversion to succinyl coenzyme A; a reaction necessary in the breakdown of fats and amino acids [7]. This leads to an accumulation of methylmalonic acid and proprionic acid. Both are abnormal fatty acids substrates which are incorporated into the myelin sheath. A summary of the key elements of this biochemical are summarized in Table 2.

**Table 2:** A summary of key biochemical contributors to the nitrous oxide induced methyl folate trap

Enzyme/cofactor	Mechanism of action	Effects of Inhibition	Serological marker
Cobalt	Cofactor of methionine synthetase	Deactivates cobalamin	None
Methionine synthase	Converts homocysteine to methionine	Failure to methylate myelin sheaths	Elevated homocysteine
Methylenetetrahydrofolate reductase	Demethylates methyltetrahydrofolate	Methylation of deoxyuridine to deoxythymidine (DNA)	None
Methylmalonyl coenzyme A mutase	Methylmalonyl coenzyme A conversion to succinyl coenzyme A	Buildup of fatty acids and amino acids	Elevated methylmalonic acid

**Figure 2:**
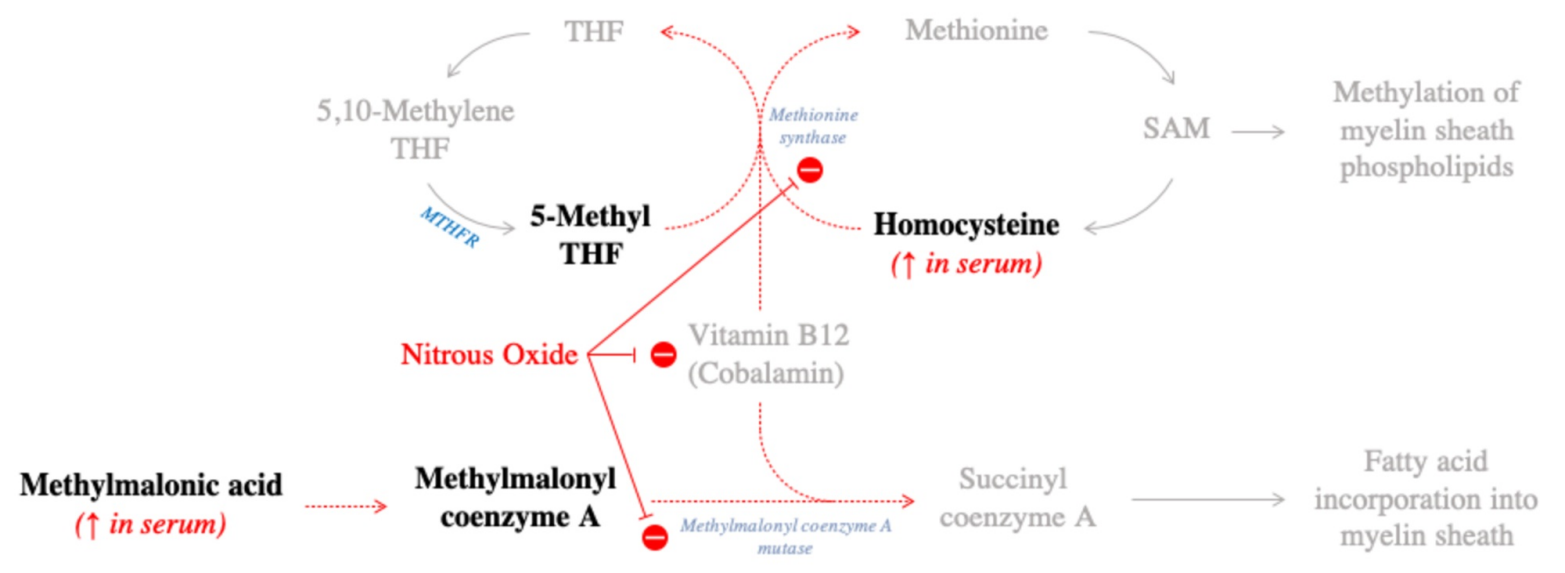
Biochemical mechanisms of nitrous oxide induced demyelination Biochemical pathways depicting the consequences of nitrous oxide induced vitamin B12 deficiency leading to elevated serum levels of methylmalonic acid and homocysteine. THF = tetrahydrofolate; SAM = S-adenosyl methionine

A visual representation of these biochemical pathways is depicted in Figure 2.

It should be noted in Figure 2, that the enzyme 5,10-methylenetetrahydrofolate reductase (MTHFR) supplies the substrate 5-methyltetrahydrofolate (5-methyl THF) for the action of MS. Studies have indicated that certain polymorphisms of MTHFR may predispose to the development of nitrous oxide neurotoxicity. Patients who are homozygous for MTHFR 677 C to T or 1298 A to C develop significantly higher homocysteine levels after nitrous anesthesia than non-carriers [3,4]. Our patient’s symptoms worsened after initiation of 5 mg mega doses of vitamin B12 despite normal folate levels. The biochemical reason for this has been well documented in the literature pertaining to subacute combined degeneration of the spinal cord secondary to vitamin B12 deficiency with worsening of symptoms due to the methyl folate trap [5,6]. As seen in Figure 2, the inhibition of MS leads to accumulation of 5-methyl THF which cannot be metabolized by MS or converted to 5,10-methylene THF, the active methyl donor form. In the inactive form, 5-methyl THF is functionally useless. This explains the patient’s worsening symptoms following vitamin B12 administration despite normal serum folate levels. Fortunately, this trap can be overcome by administering synthetic folic acid which is converted directly into dihydrofolate and subsequently into THF and 5,10-methylene THF [8]. This pathway replenishes the supply of 5,10-methylene THF, thereby reactivating the methyl donor involved in myelination.

## Conclusions

Posterior cord myelopathy is a well-known complication of prolonged nitrous oxide inhalation resulting in low or dysfunctional vitamin B12. In this case, we document a patient with a nitrous oxide substance use disorder who deteriorated following a corrective treatment with vitamin B12 supplementation and in the presence of normal serum vitamin B12 and folate levels. Exacerbation of his symptoms implicated the presence of a methyl folate trap, which was bypassed by nutritional folate supplementation. While the phenomenon of the methyl folate trap has been recognized in cases of pernicious anemia-induced subacute combined degeneration of the spinal cord, the knowledge and understanding of this phenomenon should be applied to include all forms of vitamin B12 deficiency. This includes prolonged nitrous oxide use, since the metabolic pathways outlined here are a common denominator for all etiologies of vitamin B12 deficiency, both qualitatively and quantitatively. This case highlights the importance of providing folate with vitamin B12 supplementations in patients experiencing progressive neurotoxicity arising from vitamin B12 deficiency, regardless of the cause.
